# Digestive Characteristics of *Hyphantria cunea* Larvae on Different Host Plants

**DOI:** 10.3390/insects14050463

**Published:** 2023-05-14

**Authors:** Aoying Zhang, Tao Li, Lisha Yuan, Mingtao Tan, Dun Jiang, Shanchun Yan

**Affiliations:** 1 School of Forestry, Northeast Forestry University, Harbin 150040, China; aoyingzhang@nefu.edu.cn (A.Z.);; 2 Key Laboratory of Sustainable Forest Ecosystem Management-Ministry of Education, Northeast Forestry University, Harbin 150040, China

**Keywords:** *Hyphantria cunea*, digestive enzymes, nutrients, host plant, adaptation

## Abstract

**Simple Summary:**

*Hyphantria cunea* is a cosmopolitan quarantine pest that can sustain on a wide range of host plants. However, the role of the digestive physiology of *H. cunea* larvae in regulating its multi-host adaptation has not been reported. Herein, we found that α-amylase and trypsin played an important role in nutrient metabolism and host adaptation of *H. cunea*. Furthermore, *H. cunea* larvae had highly adaptable compensatory mechanisms for digestion in response to insect digestive enzyme inhibitors. The findings reveal that digestive physiology mediates the multi-host adaptation of *H. cunea* larvae.

**Abstract:**

Digestive physiology mediates the adaptation of phytophagous insects to host plants. In this study, the digestive characteristics of *Hyphantria cunea* larvae feeding preferences on different host plants were investigated. The results showed that the body weight, food utilization, and nutrient contents of *H. cunea* larvae feeding on the high-preference host plants were significantly higher than those feeding on the low-preference host plants. However, the activity of larval digestive enzymes in different host plants presented an opposite trend, as higher α-amylase or trypsin activity was observed in the group feeding on the low-preference host plants than that feeding on the high-preference host plants. Upon treatment of leaves with α-amylase and trypsin inhibitors, the body weight, food intake, food utilization rate, and food conversion rate of *H. cunea* larvae significantly decreased in all host plant groups. Furthermore, the *H. cunea* comprised highly adaptable compensatory mechanisms of digestion involving digestive enzymes and nutrient metabolism in response to digestive enzyme inhibitors. Taken together, digestive physiology mediates the adaptation of *H. cunea* to multiple host plants, and the compensatory effect of digestive physiology is an important counter-defense strategy implemented by *H. cunea* to resist plant defense factors, especially the insect digestive enzyme inhibitors.

## 1. Introduction

*Hyphantria cunea* (Lepidoptera: Erebidae) is a cosmopolitan quarantine pest that originated in North America and is mainly distributed in the United States and southern Canada [1,2]. It was first introduced in Hungary from its origin in 1940 and exploded on a large scale in 1945 [3]. At present, *H. cunea* has invaded over 32 countries [4]. In China, the *H. cunea* invasion began in 1979, and since then, it has spread to 613 county-level administrative regions in 14 Chinese provinces. *H. cunea* exhibits the characteristics of high food intake and prolonged damage time [5]. Upon invasion, *H. cunea* can eat up the whole plant leaves, inhibit the growth of trees and crops, and eventually result in severe destruction of forest and agriculture ecological balance [6]. Additionally, *H. cunea* often feeds and pupates on plants in public areas, such as parks and on the sides of roads, thereby affecting the daily life of local residents [7]. The damages caused by *H. cunea* have seriously threatened agriculture and forestry production and ecological security [8]. In 2022, ten Chinese ministerial-level departments listed the control of *H. cunea* as a key mission.

*H. cunea*, as a high polyphagy pest, can sustain on a wide range of host plants. It has been reported that *H. cunea* can feed on 636 host plants, of which 120 are found in the United States, 300 in Japan, 219 in South Korea, and 234 in Europe [9,10,11,12]. In China, more than 300 host plants have been reported as potential hosts for *H. cunea*, covering almost all the tree, flower, and crop species [13]. Phytophagous insects exhibit distinct preferences for the host plants [14]. Schowalter et al. (2017) found that *H. cunea* exhibits a high preference for *Morus alba*, *Platanus acerifolia*, *Carya cathayensis*, *Fraxinus pennsylvanica*, and *Ulmus americana* [7]. Jang et al. (2015) found that *Cornus kousa* and most coniferous tree species are non-feeding species for *H. cunea* [15]. Numerous studies revealed that during the process of co-evolution between phytophagous insects and plants, insects evolved a digestive physiological regulatory mechanism to achieve the best adaptation for different host plants. As demonstrated by Lazarevi et al. (2017), *Lymantria dispar* larvae exhibit increased trypsin activity upon feeding on the low-preference host plant, *Robinia pseudoacacia*, compared with the high-preference host plant, *Quercus palustris* [16]. Sarate et al. (2012) showed that *Helicoverpa armigera* exhibits enhanced larval adaptation to pigeon peas and chickpeas (low-preference host plants) through increasing gut amylase activity [17]. Strong host adaptability is also an important aspect of the wide host spectrum for *H. cunea* [18]. Currently, the studies on the host adaptability of *H. cunea* mainly focus on the host plant screening, as well as the feeding and oviposition selection behavior of *H. cunea* to different host plants [19,20]. However, no systematic study has been conducted to explore the adaptive mechanism of *H. cunea* larvae to multiple host plants from the perspective of digestive physiology.

Digestion and absorption are two important aspects of digestive physiology and can affect the adaptability of phytophagous insects to host plants at varying degrees [21]. The levels of these two aspects are mainly determined by the digestive enzyme activity of phytophagous insects, such as α-amylase and trypsin. In this regard, we hypothesized that digestive physiology based on digestive enzymes mediates the formation of multiple host characteristics of *H. cunea*. To test the above-mentioned hypothesis, we fed *H. cunea* larvae with leaves of high-preference (*Ulmus pumila*, *Fraxinus mandshurica*, and *M. alba*), medium-preference (*Juglans mandshurica* and *Betula platyphylla*), and low-preference (*Albizia kalkora* and *Tilia amurensis*) host plants. Subsequently, the larval growth and food utilization status, content of nutrients, and activity of digestive enzymes were measured. Furthermore, the regulatory roles of digestive physiology on the adaptation of *H. cunea* to multiple host plants were demonstrated in reverse by the treatment of α-amylase and trypsin inhibitors. The relevant findings of this study helped in determining the correlation between the digestive physiology and multi-host adaptability of *H. cunea* and provide a theoretical foundation to analyze the outbreak of *H. cunea*.

## 2. Materials and Methods

### 2.1. Experimental Host Plants

According to the results of our previous study [22], the larvae of H. cunea exhibit a high preference for *U. pumila* (UP), *F. mandshurica* (FM), and *M. alba* (MA), a medium preference for *J. mandshurica* (JM) and *B. platyphylla* (BP), and a low preference for *A. kalkora* (AK) and *T. amurensis* (TA). Therefore, these seven tree species were selected as host plants to test in this study. Except for MA, which was 2 years old (purchased from Baima Mountain Spring Agricultural Development Co., Ltd., Taian, China), the rest of the trees were 3 years old (purchased from Shenyang Ruihe Technology Service Co., Ltd., Shenyang, China) at the time of testing. In May 2022, all plants were planted in pots (400 × 350 mm) containing 10 kg of soil substrate. The soil substrate consisted of sand, turf soil, and vermiculite at a ratio of 1:3:1. A total of 300 host plants per species were planted and maintained under natural light conditions. During the experiment, the plants were watered every two days and weeded over time to ensure normal growth.

### 2.2. Experimental Insects

In July 2022, egg masses of *H. cunea* were collected from Hengshui, Hebei province, China. The egg masses were incubated in an incubator (HPG-280HX, Harbin Donglian Electronic Technology Development Co., Ltd., Harbin, China) at a temperature of 25 ± 1 °C, relative humidity of 70 ± 1%, and a photoperiod of 16L:8D [23]. After hatching, the larvae were reared on the artificial diets (purchased from the Ecology and Nature Conservation Institute, Chinese Academy of Forestry, Beijing, China) under the same conditions until the third instar. Third-instar larvae with a molting time of less than 24 h were selected. All larvae were divided into 7 groups and reared on the leaves of 7 host plants, respectively. The temperature, humidity, and photoperiod of the larval growth environment were consistent with the incubation environment. The larvae were not starved before being transferred from artificial diet to the leaves. The larvae were kept in plastic boxes that contain sufficient leaves (133 × 80 × 48 mm). Each box was fed with 5 larvae. To keep the food plentiful and fresh, the leaves were changed once a day.

### 2.3. Treatment with Insect Digestive Enzyme Inhibitors

Newly molted 4th instar larvae were selected from each host plant group. These larvae from each host plant group were divided into three parts. One part was used as the non-inhibited group (denoted as CK) and continued to be reared on the original plant leaves. The other two parts were fed with leaves of host plants sprayed with either α-amylase inhibitor (denoted as AI) or trypsin inhibitor (denoted as TI), respectively. The α-amylase inhibitor was purchased from Shanghai Renbang Pharmaceutical Technology Co., Ltd., Shanghai, China and the trypsin inhibitor was purchased from Shanghai Beyotime Biotechnology (Shanghai, China). Based on the results of our preliminary experiments, the α-amylase inhibitor and trypsin inhibitor were constituted in distilled water at a concentration of 750 g/L and 200 g/L, respectively. The prepared inhibitor solutions were evenly sprayed onto the leaf surface of each host plant. A total of 1 mL of treatment solution was sprayed on every 5 leaves. After drying at room temperature, the leaves were used to feed the 4th instar larvae of *H. cunea*. When these larvae reached the 5th instar stage, they were divided into two parts. One part was propagated for the next 48 h and then collected to obtain the whole larval body or midgut tissue samples. The other part was used to determine the larval body weight and food utilization parameters.

### 2.4. Body Weight and Food Utilization of H. cunea Larvae

Using the method developed by Gao et al. (2022) and Jiang et al. (2018), the food utilization by *H. cunea* larvae was measured [24,25]. During the experiment, the larval body weight and fresh leaf mass were measured before feeding, and then, the larval body weight, fecal mass, and residual leaf mass were measured after 48 h of feeding. The corrected water loss rate was calculated using the leaves that were not fed by *H. cunea* larvae. A total of three replicates were set for each group, and each replicate consisted of 10 larvae. The food intake, food consumption rate, conversion rate, and utilization rate of *H. cunea* larvae were calculated using the following formula:

Food intake (g) = (mass of fresh leaves before feeding − mass of residual leaves after feeding)/(1 − corrected water loss rate);

Food consumption rate (%) = (food intake − fecal discharge quality)/food intake × 100%;

Food conversion rate (%) = (weight after feeding − weight before feeding)/(food intake − feces discharge quality) × 100%;

Food utilization rate (%) = (weight after feeding − weight before feeding)/food intake × 100%.

### 2.5. Determination of Nutrient Contents in H. cunea Larvae

The contents of total protein (A045-4), total amino acids (A026-1-1), glucose (A54-1-1), trehalose (A49-1-1), and free fatty acids (A042-2-1) in the larvae of *H. cunea* were determined using the kit. All kits were purchased from Nanjing Jiancheng Bioengineering Institute. Briefly, the larvae in each group, at a ratio of weight (g): volume (mL) = 1:9, were mixed with normal saline and ground using a mortar and pestle. The larvae tissues for trehalose determination were ground with the extracting solution provided in the kit. The homogenate was centrifuged at the speed and time per the manufacturer’s instructions (10 min at 2500 rpm for total protein, glucose and free fatty acids; 10 min at 3500 rpm for total amino acids; 10 min at 8000× *g* for trehalose). Subsequently, the supernatant was collected for nutrient content determination according to the manufacturer’s instructions. Three replicates were set for each group, and each replicate consisted of three larvae. The total protein, total amino acids, glucose, trehalose, and free fatty acids contents of *H. cunea* were expressed as mg/g, μmol/mg prot, mmol/g prot, mg/g tissue, and mmol/g prot, respectively.

### 2.6. Determination of Digestive Enzyme Activity in the Midgut of H. cunea Larvae

The content or activity of the protein (A045-4), α-amylase (C06-1-1), and trypsin (A080-2) in *H. cunea* larval midgut were determined using the kit. All kits were purchased from Nanjing Jiancheng Bioengineering Institute. The tissue was mixed with normal saline at a ratio of weight (g): volume (mL) = 1:9. Subsequently, the samples were ground using a plastic grinding pestle in a 1.5 mL centrifuge tube. After centrifugation at 2500 rpm for 10 min, the supernatant was collected. The protein concentration and α-amylase and trypsin activities of the supernatant were determined according to the manufacturer’s instructions. Three replicates were set for each group, and each replicate consisted of midgut tissue from five larvae. Both α-amylase and trypsin activities were expressed as U/mg prot.

### 2.7. Statistical Analysis

The significance of larval body weight, food utilization index, nutrient content index, and activity of digestive enzymes among different host plant groups were analyzed by the one-way analysis of variance (ANOVA) after testing for variance homogeneity and normal distribution or achieving variance homogeneity and normal distribution by log-transforming if necessary (Tables S1 and S2). Independent sample *t*-test analysis was performed to analyze the significance of each parameter between the non-inhibited and AI treatment groups or between the non-inhibited and TI treatment groups (*p* < 0.05) (Table S3).

## 3. Results

### 3.1. Digestive Enzyme Activity of H. cunea Larvae

**Figure 1:**
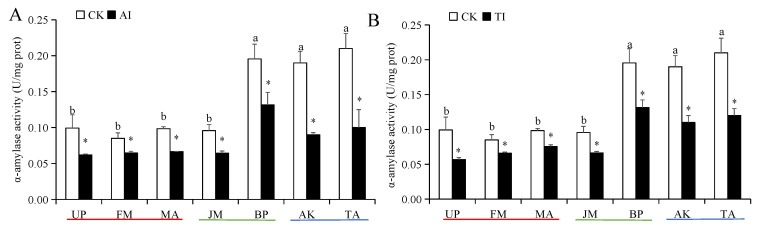
The α-amylase activities of *H. cunea* larvae treated with AI (**A**) or TI (**B**). Data in the figure are presented as the mean ± *SE* (*N* = 3); lowercase letters indicate the difference between different host plant control groups (*p* < 0.05); * indicates the significant difference between the non-inhibited and AI treatment groups or between the non-inhibited and TI treatment groups for the same plants (*p* < 0.05).

**Figure 2:**
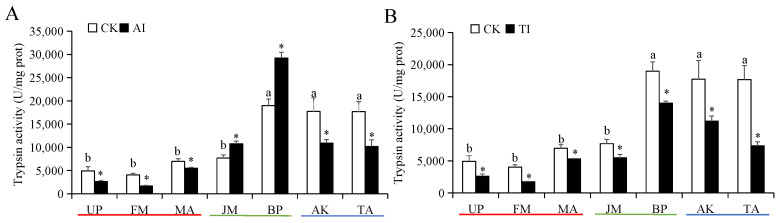
The trypsin activities of *H. cunea* larvae treated with AI (**A**) or TI (**B**). Data in the figure are presented as the mean ± *SE* (*N* = 3); lowercase letters indicate the difference between different host plant control groups (*p* < 0.05); * indicates the significant difference between the non-inhibited and AI treatment groups or between the non-inhibited and TI treatment groups for the same plants (*p* < 0.05).

The larval α-amylase and trypsin activities in the BP, AK, and TA groups were significantly higher than those in the UP, FM, MA, and JM groups (Figure 1 and Figure 2). Compared with the CK group, the larval α-amylase activity in all AI-treated host plant groups was significantly decreased (Figure 1A). Among them, those feeding on AI-treated FM groups exhibited the lowest activity. However, the larval trypsin activity was significantly increased in the medium-preference host plant (JM and BP) groups after AI treatment and significantly decreased in the high- and low-preference host plant (UP, FM, MA, AK, and TA) groups after AI treatment (Figure 2A). After TI treatment, the α-amylase and trypsin activities of larvae were significantly inhibited in all host plant groups (Figure 1B and Figure 2B). Of which, the strongest inhibition effect on the two digestive enzyme activities was observed in the TI-treated TA group, and the inhibition rate of α-amylase activity and trypsin activity was 52.38% and 58.42%, respectively (Figure 1B and Figure 2B).

### 3.2. Growth status of H. cunea Larvae 

**Figure 3:**
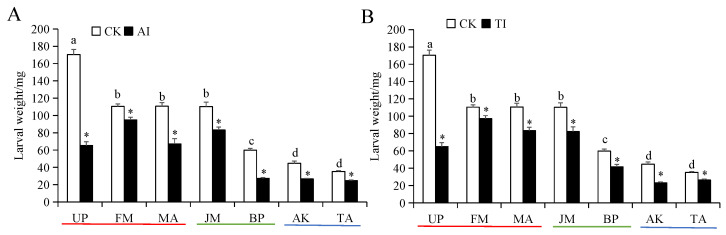
Body weight of *H. cunea* larvae treated with AI (**A**) or TI (**B**). Note: Data in the figure are presented as the mean ± *SE* (*N* = 3); lowercase letters indicate the difference between different host plant control groups (*p* < 0.05); * indicates the significant difference between the non-inhibited and AI treatment groups or between the non-inhibited and TI treatment groups for the same plants (*p* < 0.05).

The body weight of *H. cunea* larvae that were fed on high-preference host plants was significantly higher than those fed on low-preference host plants (Figure 3). Of these, the larvae that were fed on UP exhibited the highest body weight, and those fed on TA exhibited the lowest body weight. The larval body weight in all AI- or TI-treated host plant groups was significantly suppressed compared to the CK group (Figure 3A,B). The most significant suppression effect of either AI or TI treatment was observed in the UP group, with the inhibition rates as high as 61.68% and 61.85%, respectively (Figure 3A,B).

### 3.3. Food Utilization of H. cunea Larvae

**Figure 4:**
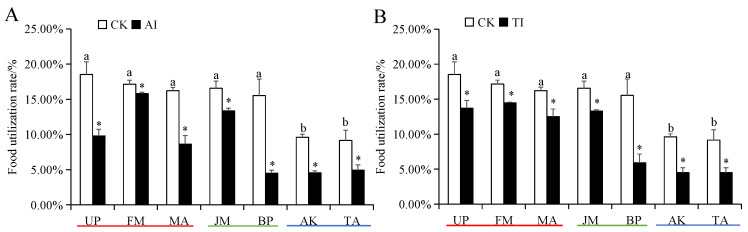
Food utilization rate of *H. cunea* larvae treated with AI (**A**) or TI (**B**). Data in the figure are presented as the mean ± *SE* (*N* = 3); lowercase letters indicate the difference between different host plant control groups (*p* < 0.05); * indicates the significant difference between the non-inhibited and AI treatment groups or between the non-inhibited and TI treatment groups for the same plants (*p* < 0.05).

**Figure 5:**
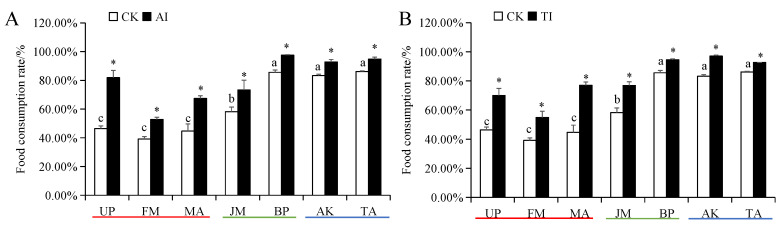
Food consumption rate of *H. cunea* larvae treated with AI (**A**) or TI (**B**). Data in the figure are presented as the mean ± *SE* (*N* = 3); lowercase letters indicate the difference between different host plant control groups (*p* < 0.05); * indicates the significant difference between the non-inhibited and AI treatment groups or between the non-inhibited and TI treatment groups for the same plants (*p* < 0.05).

The food intake and food conversion rates of *H. cunea* larvae in the high-preference host plant (UP, FM, and MA) groups were significantly higher than those in medium- and low-preference host plant groups (Figures S1 and S2). The food utilization rate of *H. cunea* larvae on high- and medium-preference host plants was significantly higher than those on low-preference host plants (Figure 4). The food consumption rate of *H. cunea* larvae on high-preference plants was significantly lower than those on medium- and low-preference host plants (Figure 5). Compared with the CK group, after UP, FM, MA, JM, BP, AK, and TA groups were treated with AI or TI, the larval food intake, food conversion rate and food utilization rate of *H. cunea* larvae exhibited a significant decrease (Figures S1 and S2 and Figure 4), whereas the larval food consumption rate was significantly elevated (Figure 5).

### 3.4. Nutrient Contents of H. cunea Larvae

The contents of various nutrients (total protein, total amino acids, glucose, trehalose, and free fatty acids) in larvae that were fed on the high-preference host plants were significantly higher than those fed on the low-preference host plants (Table 1). Upon AI or TI treatment, the total protein content of larvae significantly increased in the AK and medium-preference host plant groups (JM and BP) but significantly decreased in the high-preference host plant groups; the total amino acids content of larvae was significantly increased in the MA group, but significantly decreased in the FM, medium- and low-preference host plant groups (JM, BP, AK, and TA); the larval contents of glucose and trehalose in all plant groups were significantly decreased; the larval content of free fatty acids was significantly increased in the JM and high-preference host plant groups (UP, FM, and MA), but significantly decreased in the BP and low-preference host plant groups (AK and TA).

## 4. Discussion

Generally, phytophagous insects use their own digestive physiological mechanisms to improve the efficiency of food utilization for adapting to the host plants [26,27]. In the present study, the body weight, food utilization rate, and nutrient contents (e.g., total protein and total amino acids) of *H. cunea* larvae feeding on the high-preference host plants were found to be significantly higher than those feeding on the low-preference host plants. This supports the results of our previous study [22], indicating that *H. cunea* exhibits different preferences for the host plants. It is worth mentioning that the food consumption rate of *H. cunea* larvae in the low-preference host plant group was significantly higher than those in the high-preference host plant group. The food consumption rate is an important indicator that characterizes the food absorption capacity of phytophagous insects [25,28]. It indicates that *H. cunea* enhances the level of larval adaptation to the low-preference host plants by increasing the absorption capacity. However, inconsistent with the expected results, the α-amylase and trypsin activities in the midgut of *H. cunea* larvae feeding on the high-preference host plants were significantly lower than those feeding on the low-preference host plants. A possible reason is that *H. cunea* larvae are more easily able to digest and absorb the high-preference host plants than the low-preference host plants. Namely, it only needs relatively few digestive enzymes to efficiently absorb the nutrients from the high-preference host plants. Similarly, Chai et al. (2016) found that the amylase and cellulase activities in the gut of *Agasicles hygrophila* larvae were higher when feeding on the non-palatable host plants than those feeding on the palatable host plants [29]. A reverse trend was also reported by Shen et al. (2022), who found that among the five host plant groups, *Dendrolimus houi* larvae feeding on the high-preference host plants exhibited the highest digestive enzyme activity [30]. Our results, together with these previous findings, suggest that the response of digestive enzymes in phytophagous insects to the host plants with different preferences varies depending on the insect species or the content of endogenous inhibitors in these plants.

To further clarify the physiological function of digestive enzymes, we perturbed the digestion physiology of *H. cunea* larvae by spraying the inhibitors of digestive enzymes onto the leaf surface and then analyzed the adaptability of *H. cunea* to different host plants. Our results revealed that the body weight, food utilization status, several nutrients content, and α-amylase activity of *H. cunea* larvae upon AI treatment were significantly reduced. The function of α-amylase is to convert the starch into oligosaccharides, a precursor for glucose abortion [31,32,33]. These results suggest that the absence of α-amylase or functional impairment of α-amylase inhibits normal nutritional metabolism and attenuates the growth of *H. cunea* larvae. Trypsin is a key enzyme involved in the process of food protein hydrolysis [34]. The inhibition analysis revealed that the growth and digestive physiology trends of *H. cunea* larvae in the TI treatment group were consistent with those in the AI treatment group. It further indicates that the absence of trypsin may lead to inefficient uptake and utilization of nutrients (e.g., total protein) from the host plants by *H. cunea*, which ultimately leads to the growth retardation of *H. cunea* larvae. In addition to digestive physiology disorders, another possible reason for the reduced growth of *H. cunea* larvae caused by AI and TI is that inhibitor treatment reduces the palatability of plant leaves, as inhibitor-treated larvae in all host plant groups consume significantly less food. However, the food consumption rate increased following the addition of inhibitors across the board, irrespective of high and low preference towards host plants. This suggests once again that *H. cunea* larvae may be trying to adapt to adverse environmental conditions by improving their absorption capacity. This ability has also been reported in *H. cunea* larvae adaptation to other stressors, such as secondary metabolites and heavy metals stress [35,36,37]. Interestingly, treatment with digestive enzyme inhibitors attenuated differences in larval adaptation between high- and low-preference host plant groups, as shown by the fact that the AI and TI treatment reduced the difference in larval body weight from 65.77–135.17 mg to 38.66–74.14 mg between high and low feeding groups. A large number of studies have shown that compared with low-preference host plants, high-preference host plants have higher nutrient content and lower endogenous digestive enzyme inhibitor content [38,39,40]. After treatment with exogenous enzyme inhibitors, the *H. cunea* larvae could not effectively absorb nutrients from plants with high preference. This has led to a narrowing of the differences in the degree of adaptation. Another reason may be that digestive disorders lead to weakened digestive and taste nervous system functions, but further research is needed. Taken together, our results demonstrate that α-amylase and trypsin play an important role in nutrient metabolism and host adaptation of *H. cunea*.

Numerous studies have demonstrated that phytophagous insects are capable of regulating their digestive physiology to meet nutrient requirements [41,42,43]. This is a strategy used by insects to adapt to adverse conditions [44,45]. In the present study, the α-amylase activity in larval midgut was found to be significantly reduced in all AI-treated host plant groups, but the trypsin activity was significantly increased in the AI-treated JM and BP groups. Similar compensatory effects of digestive enzymes have been reported in other Lepidopteran insects. For example, Bezerra et al. (2017) found that *Ephestia kuehniella* exhibited a significant increase in chymotrypsin activity in vivo after treatment with TI [46]. Kuwar et al. (2019) also found that the trypsin activity of *H. armigera* larvae that were fed on soybean upon TI treatment was significantly increased [47]. These findings suggest that if the host plant has a high content of an insect digestive enzyme inhibitor, phytophagous insects can still regulate the activities of other digestive enzymes to compensate for the adverse effects of such inhibitors to promote the absorption and utilization of food nutrients [48]. Additionally, the nutrient content analysis revealed similar compensatory trends in the nutrient content of *H. cunea* larvae after treatment with AI or TI. For example, the total amino acids and free fatty acids contents were significantly higher in both AI- and TI-treated MA groups. It is further shown that *H. cunea* larvae have highly adaptable compensatory mechanisms for digestion, including digestive enzymes and nutrient metabolism, in response to unfavorable conditions. The insect digestive enzyme inhibitors secreted by plants are a common class of chemical defense compounds against phytophagous insects [47,49]. The compensatory effect exhibited by *H. cunea* to the digestive enzyme inhibitors may be implicated as a counter-defense strategy in response to their endogenous plant resistance factors. It is not difficult to conclude that the highly adaptable digestive physiological compensation effect may be the reason for the multi-host adaptability and host expansion of *H. cunea* larvae.

## 5. Conclusions

The digestive physiology of *H. cunea* plays an integral role in its adaptation to multiple host plants. The *H. cunea* larvae can improve larval food utilization and absorption efficiency on host plants by regulating the activity of larval digestive enzymes. This helps *H. cunea* maximize its adaptation to a variety of host plants and ensure its spread and host expansion. Additionally, *H. cunea* exhibits a highly adaptable digestive physiological compensation effect involving digestive enzymes and nutrient metabolism. This is implicated as a counter-defense strategy evolved by *H. cunea* in response to the endogenous resistance factors of host plants, especially the inhibitors of insect digestive enzymes. The findings of this study provide new insights into potential strategies to analyze the spread and host expansion of *H. cunea*.

## Figures and Tables

**Table 1:** Nutrients content of *H. cunea* larvae.

Nutrient Content		UP	FM	MA	JM	BP	AK	TA
Total protein (mg/g)	CK	10.53 ± 0.65 ab	11.55 ± 0.41 ab	10.02 ± 0.25 b	12.11 ± 0.41 a	7.37 ± 0.63 c	7.66 ± 0.66 c	7.08 ± 0.88 c
	AI	8.19 ± 0.18 *	9.60 ± 0.47 *	7.33 ± 0.61 *	14.83 ± 0.20 *	13.77 ± 1.24 *	10.73 ± 0.27 *	8.18 ± 0.53
	TI	7.70 ± 0.75 *	9.12 ± 0.54 *	8.08 ± 0.60 *	17.05 ± 0.66 *	14.02 ± 1.11 *	11.32 ± 1.14 *	8.63 ± 0.30
Total amino acids (μmol/mg prot)	CK	1.92 ± 0.19 a	1.90 ± 0.12 a	1.80 ± 0.05 ab	1.83 ± 0.13 ab	1.52 ± 0.19 abc	1.41 ± 0.13 c	1.18 ± 0.05 c
	AI	2.31 ± 0.20 *	1.20 ± 0.07 *	2.00 ± 0.01 *	1.09 ± 0.14 *	0.85 ± 0.11 *	0.96 ± 0.01 *	1.02 ± 0.01 *
	TI	1.94 ± 0.19	1.26 ± 0.14 *	2.30 ± 0.04 *	1.07 ± 0.02 *	0.96 ± 0.07 *	0.84 ± 0.10 *	0.86 ± 0.03 *
Glucose (μmol/g prot)	CK	110.30 ± 3.41 a	120.90 ± 11.36 a	110.90 ± 3.31 a	82.20 ± 15.37 b	55.20 ± 1.56 b	62.00 ± 6.61 b	67.60 ± 3.80 b
	AI	40.70 ± 1.97 *	85.50 ± 1.22 *	88.90 ± 4.83 *	48.10 ± 3.68 *	27.80 ± 1.74 *	41.70 ± 7.66 *	37.70 ± 1.50 *
	TI	62.40 ± 3.06 *	76.80 ± 7.96 *	81.95 ± 4.97 *	43.50 ± 6.60 *	36.90 ± 6.09 *	52.60 ± 1.57 *	53.90 ± 1.80 *
Trehalose (mg/g tissue)	CK	8.69 ± 0.30 a	9.26 ± 0.29 b	7.23 ± 0.46 c	5.75 ± 0.13 d	2.50 ± 0.21 e	1.24 ± 0.11 f	3.82 ± 0.41 g
	AI	2.43 ± 0.12 *	4.50 ± 0.52 *	1.81 ± 0.16 *	3.60 ± 0.44 *	0.74 ± 0.35 *	0.53 ± 0.15 *	0.60 ± 0.18 *
	TI	2.48 ± 0.24 *	4.21 ± 0.37 *	3.50 ± 0.28 *	3.34 ± 0.58 *	0.85 ± 0.57 *	0.38 ± 0.04 *	0.78 ± 0.23 *
Free fatty acids (μmol/g prot)	CK	114.60 ± 11.28 a	55.00 ± 3.40 b	105.60 ± 7.98 a	49.00 ± 11.30 b	6.30 ± 1.28 c	11.20 ± 1.60 c	9.40 ± 2.00 c
	AI	152.90 ± 6.38 *	79.40 ± 11.24 *	223.90 ± 6.15 *	80.20 ± 5.09 *	1.70 ± 0.21 *	2.60 ± 0.35 *	3.10 ± 0.54 *
	TI	157.20 ± 13.98 *	79.20 ± 7.09 *	269.80 ± 9.98 *	84.10 ± 8.50 *	2.80 ± 0.91 *	2.80 ± 1.94 *	3.10 ± 1.58 *

Data in the table are presented as the mean ± *SE* (*N* = 3); lowercase letters indicate the difference between different host plant control groups (*p* < 0.05); * Indicates the significant difference between the non-inhibited and AI treatment groups or between the non-inhibited and TI treatment groups for the same nutrients in the same plants (*p* < 0.05).

## Data Availability

The data presented in this study are available on request from the corresponding author.

## Supplementary Materials

The following supporting information can be downloaded at: https://www.mdpi.com/article/10.3390/insects14050463/s1; Figure S1: Food intake of *H. cunea* larvae; Figure S2: Food conversion rate of *H. cunea* larvae; Table S1: Multiple comparisons; Table S2: Test of homogeneity of variances; Table S3: Independent samples test.
