# Supplementary material for: Digestive Characteristics of Hyphantria cunea Larvae on Different Host Plants
Source: Insects. 2023 May 14;14(5):463. doi: 10.3390/insects14050463 (PMC10231093; doi:10.3390/insects14050463)
Supplement: Supplementary file 1 [file insects-14-00463-s001.zip › insects-2350829-supplementary.pdf]

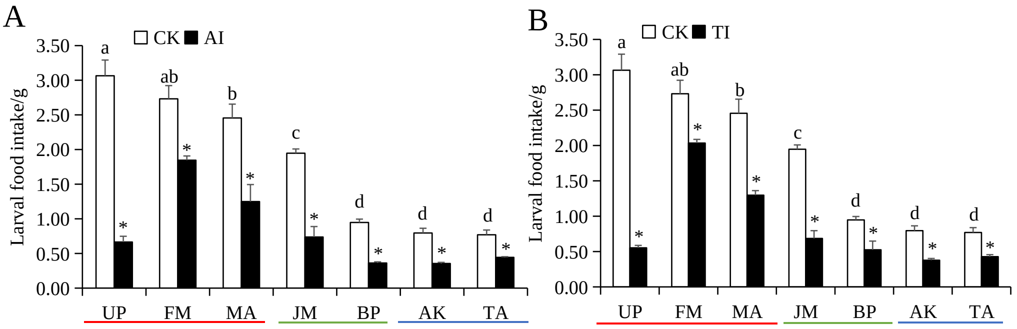

Figure S1. Food intake of *H. cunea* larvae treated with AI (**A**) or TI (**B**). Data in the figure are presented as the mean ± *SE* (*N*=3); lower case letters indicate the difference between different host plant control groups (*P*<0.05); * indicates the significant difference between the non-inhibited and AI treatment groups or between the non-inhibited and TI treatment groups for the same plants (*P*<0.05).

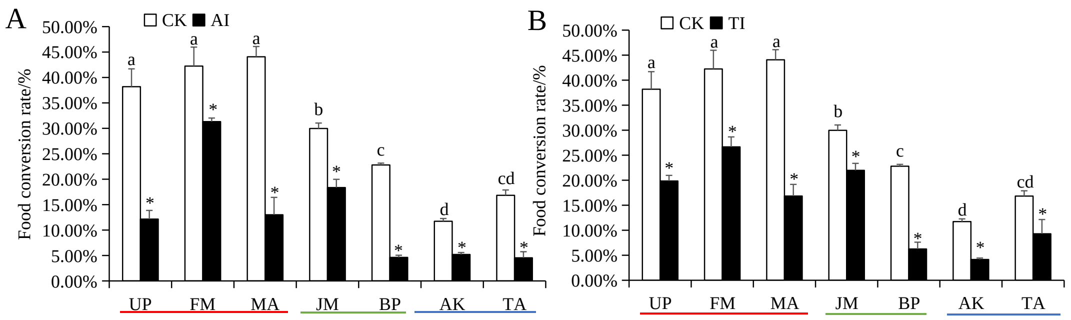

Figure S2. Food conversion rate of *H. cunea* larvae treated with AI (**A**) or TI (**B**). Data in the figure are presented as the mean ± *SE* (*N*=3); lower case letters indicate the difference between different host plant control groups ($P<0.05$); * indicates the significant difference between the non-inhibited and AI treatment groups or between the non-inhibited and TI treatment groups for the same plants ($P<0.05$).

Table S1. Multiple comparisons.

Dependent Variable	(I) Groups	(J) Groups	Sig.
α-amylase activity	UP	FM	0.512
		MA	0.963
		JM	0.866
		BP	0
		AK	0.001
		TA	0
	FM	UP	0.512
		MA	0.541
		JM	0.624
		BP	0
		AK	0
		TA	0
	MA	UP	0.963
		FM	0.541
		JM	0.902
		BP	0
		AK	0.001
		TA	0
	JM	UP	0.866
		FM	0.624
		MA	0.902
		BP	0
		AK	0.001
		TA	0
	BP	UP	0
		FM	0
		MA	0
		JM	0
		AK	0.782
		TA	0.512
	AK	UP	0.001
		FM	0
		MA	0.001
		JM	0.001
		BP	0.782
		TA	0.356
	TA	UP	0
		FM	0
		MA	0
		JM	0
		BP	0.512
		AK	0.356

Trypsin activity	UP	FM	0.695
		MA	0.371
		JM	0.233
		BP	0
		AK	0
		TA	0
	FM	UP	0.695
		MA	0.207
		JM	0.122
		BP	0
		AK	0
		TA	0
	MA	UP	0.371
		FM	0.207
		JM	0.752
		BP	0
		AK	0
		TA	0
	JM	UP	0.233
		FM	0.122
		MA	0.752
		BP	0
		AK	0
		TA	0
	BP	UP	0
		FM	0
		MA	0
		JM	0
		AK	0.585
		TA	0.569
	AK	UP	0
		FM	0
		MA	0
		JM	0
		BP	0.585
		TA	0.981
	TA	UP	0
		FM	0
		MA	0
		JM	0
		BP	0.569
		AK	0.981
Larval weight	UP	FM	0
		MA	0

		JM	0
		BP	0
		AK	0
		TA	0
	FM	UP	0
		MA	0.982
		JM	0.982
		BP	0
		AK	0
		TA	0
	MA	UP	0
		FM	0.982
		JM	0.964
		BP	0
		AK	0
		TA	0
	JM	UP	0
		FM	0.982
		MA	0.964
		BP	0
		AK	0
		TA	0
	BP	UP	0
		FM	0
		MA	0
		JM	0
		AK	0.104
		TA	0.013
	AK	UP	0
		FM	0
		MA	0
		JM	0
		BP	0.104
		TA	0.295
	TA	UP	0
		FM	0
		MA	0
		JM	0
		BP	0.013
		AK	0.295
Food intake	UP	FM	0.122
		MA	0.009
		JM	0
		BP	0

		AK	0
		TA	0
	FM	UP	0.122
		MA	0.189
		JM	0.002
		BP	0
		AK	0
		TA	0
	MA	UP	0.009
		FM	0.189
		JM	0.026
		BP	0
		AK	0
		TA	0
	JM	UP	0
		FM	0.002
		MA	0.026
		BP	0
		AK	0
		TA	0
	BP	UP	0
		FM	0
		MA	0
		JM	0
		AK	0.46
		TA	0.387
	AK	UP	0
		FM	0
		MA	0
		JM	0
		BP	0.46
		TA	0.897
	TA	UP	0
		FM	0
		MA	0
		JM	0
		BP	0.387
		AK	0.897
Food conversion rate	UP	FM	0.208
		MA	0.076
		JM	0.018
		BP	0
		AK	0
		TA	0

	FM	UP	0.208
		MA	0.562
		JM	0.001
		BP	0
		AK	0
		TA	0
	MA	UP	0.076
		FM	0.562
		JM	0
		BP	0
		AK	0
		TA	0
	JM	UP	0.018
		FM	0.001
		MA	0
		BP	0.035
		AK	0
		TA	0.001
	BP	UP	0
		FM	0
		MA	0
		JM	0.035
		AK	0.003
		TA	0.072
	AK	UP	0
		FM	0
		MA	0
		JM	0
		BP	0.003
		TA	0.12
	TA	UP	0
		FM	0
		MA	0
		JM	0.001
		BP	0.072
		AK	0.128
Food utilization rate	UP	FM	0.437
		MA	0.251
		JM	0.884
		BP	0.102
		AK	0
		TA	0.001
	FM	UP	0.437
		MA	0.697

		JM	0.525
		BP	0.358
		AK	0.001
		TA	0.003
	MA	UP	0.251
		FM	0.697
		JM	0.312
		BP	0.59
		AK	0.001
		TA	0.008
	JM	UP	0.884
		FM	0.525
		MA	0.312
		BP	0.132
		AK	0
		TA	0.001
	BP	UP	0.102
		FM	0.358
		MA	0.59
		JM	0.132
		AK	0.004
		TA	0.022
	AK	UP	0
		FM	0.001
		MA	0.001
		JM	0
		BP	0.004
		TA	0.382
	TA	UP	0.001
		FM	0.003
		MA	0.008
		JM	0.001
		BP	0.022
		AK	0.382
Food consumption rate	UP	FM	0.469
		MA	0.659
		JM	0.007
		BP	0
		AK	0
		TA	0
	FM	UP	0.469
		MA	0.774
		JM	0.002
		BP	0

		AK	0
		TA	0
	MA	UP	0.659
		FM	0.774
		JM	0.003
		BP	0
		AK	0
		TA	0
	JM	UP	0.007
		FM	0.002
		MA	0.003
		BP	0
		AK	0
		TA	0
	BP	UP	0
		FM	0
		MA	0
		JM	0
		AK	0.554
		TA	0.884
	AK	UP	0
		FM	0
		MA	0
		JM	0
		BP	0.554
		TA	0.463
	TA	UP	0
		FM	0
		MA	0
		JM	0
		BP	0.884
		AK	0.463
Total protein	UP	FM	0.241
		MA	0.553
		JM	0.079
		BP	0.002
		AK	0.004
		TA	0.001
	FM	UP	0.241
		MA	0.088
		JM	0.516
		BP	0
		AK	0
		TA	0

	MA	UP	0.553
		FM	0.088
		JM	0.025
		BP	0.007
		AK	0.014
		TA	0.003
	JM	UP	0.079
		FM	0.516
		MA	0.025
		BP	0
		AK	0
		TA	0
	BP	UP	0.002
		FM	0
		MA	0.007
		JM	0
		AK	0.732
		TA	0.732
	AK	UP	0.004
		FM	0
		MA	0.014
		JM	0
		BP	0.732
		TA	0.496
	TA	UP	0.001
		FM	0
		MA	0.003
		JM	0
		BP	0.732
		AK	0.496
Total amino acids	UP	FM	0.912
		MA	0.518
		JM	0.665
		BP	0.055
		AK	0.018
		TA	0.002
	FM	UP	0.912
		MA	0.59
		JM	0.746
		BP	0.068
		AK	0.023
		TA	0.002
	MA	UP	0.518
		FM	0.59

		JM	0.828
		BP	0.174
		AK	0.065
		TA	0.006
	JM	UP	0.665
		FM	0.746
		MA	0.828
		BP	0.121
		AK	0.043
		TA	0.004
	BP	UP	0.055
		FM	0.068
		MA	0.174
		JM	0.121
		AK	0.575
		TA	0.096
	AK	UP	0.018
		FM	0.023
		MA	0.065
		JM	0.043
		BP	0.575
		TA	0.247
	TA	UP	0.002
		FM	0.002
		MA	0.006
		JM	0.004
		BP	0.096
		AK	0.247
Glucose	UP	FM	0.4
		MA	0.963
		JM	0.037
		BP	0
		AK	0.001
		TA	0.003
	FM	UP	0.4
		MA	0.425
		JM	0.007
		BP	0
		AK	0
		TA	0.001
	MA	UP	0.963
		FM	0.425
		JM	0.033
		BP	0

		AK	0.001
		TA	0.003
	JM	UP	0.037
		FM	0.007
		MA	0.033
		BP	0.043
		AK	0.118
		TA	0.249
	BP	UP	0
		FM	0
		MA	0
		JM	0.043
		AK	0.588
		TA	0.327
	AK	UP	0.001
		FM	0
		MA	0.001
		JM	0.118
		BP	0.588
		TA	0.651
	TA	UP	0.003
		FM	0.001
		MA	0.003
		JM	0.249
		BP	0.327
		AK	0.651
Trehalose	UP	FM	0.2
		MA	0.004
		JM	0
		BP	0
		AK	0
		TA	0
	FM	UP	0.2
		MA	0
		JM	0
		BP	0
		AK	0
		TA	0
	MA	UP	0.004
		FM	0
		JM	0.004
		BP	0
		AK	0
		TA	0

	JM	UP	0
		FM	0
		MA	0.004
		BP	0
		AK	0
		TA	0
	BP	UP	0
		FM	0
		MA	0
		JM	0
		AK	0.01
		TA	0.008
	AK	UP	0
		FM	0
		MA	0
		JM	0
		BP	0.01
		TA	0
	TA	UP	0
		FM	0
		MA	0
		JM	0
		BP	0.008
		AK	0
Free fatty acids	UP	FM	0
		MA	0.385
		JM	0
		BP	0
		AK	0
		TA	0
	FM	UP	0
		MA	0
		JM	0.552
		BP	0
		AK	0.001
		TA	0
	MA	UP	0.385
		FM	0
		JM	0
		BP	0
		AK	0
		TA	0
	JM	UP	0
		FM	0.552

	MA	0
	BP	0.001
	AK	0.002
	TA	0.001
BP	UP	0
	FM	0
	MA	0
	JM	0.001
	AK	0.629
	TA	0.905
AK	UP	0
	FM	0.001
	MA	0
	JM	0.002
	BP	0.629
	TA	0.715
TA	UP	0
	FM	0
	MA	0
	JM	0.001
	BP	0.905
	AK	0.715

Table S2. Test of homogeneity of variances.

	Levene Statistic	Df1	Df2	Sig.
α-amylase activity	1.525	6	14	0.241
Trypsin activity	1.285	6	14	0.325
Larval weight	2.212	6	14	0.104
Food intake	2.318	6	14	0.091
Food conversion rate	2.409	6	14	0.082
Food utilization rate	2.629	6	14	0.064
Food consumption rate	2.255	6	14	0.099
Total protein	1.019	6	14	0.452
Total amino acids	1.506	6	14	0.247
Glucose	2.038	6	14	0.128
Trehalose	1.173	6	14	0.374
Free fatty acids	1.646	6	14	0.207

Table S3. Independent samples test.

Groups		Levene's Test for Equality of Variances		t-test for Equality of Means		
		F	Sig.	t	df	Sig. (2-tailed)
α-amylase activity-CKvsAI-UP	Equal variances assumed	10.559	0.031	5.014	4	0.007
	Equal variances not assumed			5.014	2.099	0.034
α-amylase activity-CKvsTI-UP	Equal variances assumed	5.229	0.084	5.436	4	0.006
	Equal variances not assumed			5.436	2.536	0.019
α-amylase activity-CKvsAI-FM	Equal variances assumed	0.637	0.47	5.413	4	0.006
	Equal variances not assumed			5.413	3.622	0.007
α-amylase activity-CKvsTI-FM	Equal variances assumed	2.4	0.196	5.563	4	0.005
	Equal variances not assumed			5.563	2.941	0.012
α-amylase activity-CKvsAI-MA	Equal variances assumed	4.994	0.089	12.035	4	0
	Equal variances not assumed			12.035	2	0.007
α-amylase activity-CKvsTI-MA	Equal variances assumed	0	1	6.397	4	0.003
	Equal variances not assumed			6.397	3.975	0.003
α-amylase activity-CKvsAI-JM	Equal variances assumed	1.779	0.253	3.428	4	0.027
	Equal variances not assumed			3.428	2.444	0.057
α-amylase activity-CKvsTI-JM	Equal variances assumed	2.291	0.205	3.282	4	0.03
	Equal variances not assumed			3.282	2.253	0.069
α-amylase activity-CKvsAI-BP	Equal variances assumed	1.815	0.249	2.963	4	0.041
	Equal variances not assumed			2.963	2.53	0.073

α-amylase activity-CKvsTI-BP	Equal variances assumed	0.835	0.412	2.78	4	0.05
	Equal variances not assumed			2.78	3.067	0.067
α-amylase activity-CKvsAI-AK	Equal variances assumed	3.522	0.134	6.164	4	0.004
	Equal variances not assumed			6.164	2.108	0.022
α-amylase activity-CKvsTI-AK	Equal variances assumed	0.451	0.539	4.212	4	0.014
	Equal variances not assumed			4.212	3.421	0.019
α-amylase activity-CKvsAI-TA	Equal variances assumed	0.416	0.554	3.302	4	0.03
	Equal variances not assumed			3.302	3.739	0.033
α-amylase activity-CKvsTI-TA	Equal variances assumed	2.579	0.184	3.833	4	0.019
	Equal variances not assumed			3.833	2.696	0.038
Trypsin activity-CKvsAI-UP	Equal variances assumed	3.075	0.154	3.225	4	0.032
	Equal variances not assumed			3.225	2.621	0.058
Trypsin activity-CKvsTI-UP	Equal variances assumed	1.718	0.26	3.068	4	0.037
	Equal variances not assumed			3.068	3.033	0.054
Trypsin activity-CKvsAI-FM	Equal variances assumed	13.002	0.023	6.366	4	0.003
	Equal variances not assumed			6.366	2.016	0.023
Trypsin activity-CKvsTI-FM	Equal variances assumed	10.309	0.033	5.886	4	0.004
	Equal variances not assumed			5.886	2.098	0.025
Trypsin activity-CKvsAI-MA	Equal variances assumed	3.435	0.137	2.79	4	0.049
	Equal variances not assumed			2.79	2.208	0.097
Trypsin activity-CKvsTI-MA	Equal variances assumed	4.252	0.108	3.176	4	0.034
	Equal variances not assumed			3.176	2.082	0.082

Trypsin activity-CKvsAI-JM	Equal variances assumed	0.202	0.677	-3.518	4	0.024
	Equal variances not assumed			-3.518	3.903	0.026
Trypsin activity-CKvsTI-JM	Equal variances assumed	3.354	0.141	2.981	4	0.041
	Equal variances not assumed			2.981	2.862	0.062
Trypsin activity-CKvsAI-BP	Equal variances assumed	0.706	0.448	-3.186	4	0.033
	Equal variances not assumed			-3.186	3.627	0.038
Trypsin activity-CKvsTI-BP	Equal variances assumed	7.611	0.051	3.313	4	0.03
	Equal variances not assumed			3.313	2.181	0.071
Trypsin activity-CKvsAI-AK	Equal variances assumed	1.704	0.262	4.724	4	0.009
	Equal variances not assumed			4.724	3.295	0.015
Trypsin activity-CKvsTI-AK	Equal variances assumed	1.421	0.299	4.453	4	0.011
	Equal variances not assumed			4.453	3.401	0.016
Trypsin activity-CKvsAI-TA	Equal variances assumed	1.182	0.338	2.884	4	0.045
	Equal variances not assumed			2.884	3.443	0.054
Trypsin activity-CKvsTI-TA	Equal variances assumed	6.599	0.062	4.57	4	0.01
	Equal variances not assumed			4.57	2.314	0.034
Larval weight-CKvsAI-UP	Equal variances assumed	1.503	0.287	8.311	4	0.001
	Equal variances not assumed			8.311	3.37	0.002
Larval weight-CKvsTI-UP	Equal variances assumed	13.513	0.021	9.816	4	0.001
	Equal variances not assumed			9.816	2.026	0.01
Larval weight-CKvsAI-FM	Equal variances assumed	3.245	0.146	4.333	4	0.012
	Equal variances not assumed			4.333	2.85	0.025

Larval weight-CKvsTI-FM	Equal variances assumed	7.949	0.048	2.903	4	0.044
	Equal variances not assumed			2.903	2.241	0.088
Larval weight-CKvsAI-MA	Equal variances assumed	0.793	0.424	3.581	4	0.023
	Equal variances not assumed			3.581	3.372	0.031
Larval weight-CKvsTI-MA	Equal variances assumed	1.975	0.233	3.233	4	0.032
	Equal variances not assumed			3.233	2.832	0.052
Larval weight-CKvsAI-JM	Equal variances assumed	1.907	0.239	4.211	4	0.014
	Equal variances not assumed			4.211	3.194	0.022
Larval weight-CKvsTI-JM	Equal variances assumed	3.41	0.139	4.551	4	0.01
	Equal variances not assumed			4.551	2.768	0.024
Larval weight-CKvsAI-BP	Equal variances assumed	1.922	0.238	11.03	4	0
	Equal variances not assumed			11.03	2.483	0.004
Larval weight-CKvsTI-BP	Equal variances assumed	6.649	0.061	1.665	4	0.171
	Equal variances not assumed			1.665	2.339	0.219
Larval weight-CKvsAI-AK	Equal variances assumed	6.305	0.066	2.9	4	0.044
	Equal variances not assumed			2.9	2.085	0.096
Larval weight-CKvsTI-AK	Equal variances assumed	7.366	0.053	3.487	4	0.025
	Equal variances not assumed			3.487	2.026	0.072
Larval weight-CKvsAI-TA	Equal variances assumed	5.249	0.084	8.668	4	0.001
	Equal variances not assumed			8.668	2.065	0.012
Larval weight-CKvsTI-TA	Equal variances assumed	6.563	0.063	14.571	4	0
	Equal variances not assumed			14.571	2.331	0.002

Food intake-CKvsAI-UP	Equal variances assumed	2.648	0.179	9.964	4	0.001
	Equal variances not assumed			9.964	2.528	0.004
Food intake-CKvsTI-UP	Equal variances assumed	5.218	0.084	10.982	4	0
	Equal variances not assumed			10.982	2.096	0.007
Food intake-CKvsAI-FM	Equal variances assumed	2.031	0.227	4.419	4	0.012
	Equal variances not assumed			4.419	2.405	0.034
Food intake-CKvsTI-FM	Equal variances assumed	2.423	0.195	3.526	4	0.024
	Equal variances not assumed			3.526	2.29	0.059
Food intake-CKvsAI-MA	Equal variances assumed	0.092	0.776	3.815	4	0.019
	Equal variances not assumed			3.815	3.842	0.02
Food intake-CKvsTI-MA	Equal variances assumed	5.163	0.086	5.498	4	0.005
	Equal variances not assumed			5.498	2.41	0.021
Food intake-CKvsAI-JM	Equal variances assumed	0.143	0.725	14.33	4	0
	Equal variances not assumed			14.33	3.968	0
Food intake-CKvsTI-JM	Equal variances assumed	0.893	0.398	10.036	4	0.001
	Equal variances not assumed			10.036	3.093	0.002
Food intake-CKvsAI-BP	Equal variances assumed	5.226	0.084	11.693	4	0
	Equal variances not assumed			11.693	2.495	0.003
Food intake-CKvsTI-BP	Equal variances assumed	5.186	0.085	3.219	4	0.032
	Equal variances not assumed			3.219	2.584	0.06
Food intake-CKvsAI-AK	Equal variances assumed	9.268	0.038	6.293	4	0.003
	Equal variances not assumed			6.293	2.18	0.02

Food intake-CKvsTI-AK	Equal variances assumed	5.518	0.079	5.672	4	0.005
	Equal variances not assumed			5.672	2.532	0.017
Food intake-CKvsAI-TA	Equal variances assumed	4.734	0.095	4.722	4	0.009
	Equal variances not assumed			4.722	2.127	0.037
Food intake-CKvsTI-TA	Equal variances assumed	2.027	0.228	4.628	4	0.01
	Equal variances not assumed			4.628	2.701	0.024
Food conversion rate-CKvsAI-UP	Equal variances assumed	0.815	0.418	6.676	4	0.003
	Equal variances not assumed			6.676	2.89	0.008
Food conversion rate-CKvsTI-UP	Equal variances assumed	1.711	0.261	4.981	4	0.008
	Equal variances not assumed			4.981	2.402	0.026
Food conversion rate-CKvsAI-FM	Equal variances assumed	4.487	0.102	2.873	4	0.045
	Equal variances not assumed			2.873	2.142	0.095
Food conversion rate-CKvsTI-FM	Equal variances assumed	1.132	0.347	3.68	4	0.021
	Equal variances not assumed			3.68	3.054	0.034
Food conversion rate-CKvsAI-MA	Equal variances assumed	0.837	0.412	7.823	4	0.001
	Equal variances not assumed			7.823	3.247	0.003
Food conversion rate-CKvsTI-MA	Equal variances assumed	0.003	0.959	8.834	4	0.001
	Equal variances not assumed			8.834	3.924	0.001
Food conversion rate-CKvsAI-JM	Equal variances assumed	0.58	0.489	5.984	4	0.004
	Equal variances not assumed			5.984	3.473	0.006
Food conversion rate-CKvsTI-JM	Equal variances assumed	0.462	0.534	4.549	4	0.01
	Equal variances not assumed			4.549	3.754	0.012

Food conversion rate-CKvsAI-BP	Equal variances assumed	0.159	0.711	32.406	4	0
	Equal variances not assumed			32.406	3.921	0
Food conversion rate-CKvsTI-BP	Equal variances assumed	3.482	0.135	11.76	4	0
	Equal variances not assumed			11.76	2.291	0.004
Food conversion rate-CKvsAI-AK	Equal variances assumed	0.701	0.45	9.849	4	0.001
	Equal variances not assumed			9.849	3.617	0.001
Food conversion rate-CKvsTI-AK	Equal variances assumed	2.086	0.222	12.345	4	0
	Equal variances not assumed			12.345	3.074	0.001
Food conversion rate-CKvsAI-TA	Equal variances assumed	0	0.996	7.682	4	0.002
	Equal variances not assumed			7.682	3.935	0.002
Food conversion rate-CKvsTI-TA	Equal variances assumed	3.245	0.146	2.478	4	0.068
	Equal variances not assumed			2.478	2.537	0.105
Food utilization rate-CKvsAI-UP	Equal variances assumed	0.106	0.761	6.972	4	0.002
	Equal variances not assumed			6.972	3.926	0.002
Food utilization rate-CKvsTI-UP	Equal variances assumed	0.392	0.565	3.447	4	0.026
	Equal variances not assumed			3.447	3.645	0.03
Food utilization rate-CKvsAI-FM	Equal variances assumed	2.716	0.175	2.859	4	0.046
	Equal variances not assumed			2.859	2.257	0.09
Food utilization rate-CKvsTI-FM	Equal variances assumed	3.197	0.148	4.666	4	0.01
	Equal variances not assumed			4.666	2.164	0.037
Food utilization rate-CKvsAI-MA	Equal variances assumed	0.811	0.419	13.919	4	0
	Equal variances not assumed			13.919	3.595	0

Food utilization rate-CKvsTI-MA	Equal variances assumed	0.968	0.381	6.421	4	0.003
	Equal variances not assumed			6.421	3.318	0.006
Food utilization rate-CKvsAI-JM	Equal variances assumed	1.493	0.289	6.28	4	0.003
	Equal variances not assumed			6.28	3.013	0.008
Food utilization rate-CKvsTI-JM	Equal variances assumed	0.05	0.834	3.29	4	0.03
	Equal variances not assumed			3.29	3.977	0.03
Food utilization rate-CKvsAI-BP	Equal variances assumed	6.006	0.07	4.634	4	0.01
	Equal variances not assumed			4.634	2.139	0.038
Food utilization rate-CKvsTI-BP	Equal variances assumed	1.601	0.274	3.628	4	0.022
	Equal variances not assumed			3.628	3.058	0.035
Food utilization rate-CKvsAI-AK	Equal variances assumed	0.296	0.615	9.844	4	0.001
	Equal variances not assumed			9.844	3.517	0.001
Food utilization rate-CKvsTI-AK	Equal variances assumed	0.133	0.734	10.553	4	0
	Equal variances not assumed			10.553	3.723	0.001
Food utilization rate-CKvsAI-TA	Equal variances assumed	0.839	0.412	5.275	4	0.006
	Equal variances not assumed			5.275	3.001	0.013
Food utilization rate-CKvsTI-TA	Equal variances assumed	0.339	0.592	2.985	4	0.041
	Equal variances not assumed			2.985	3.807	0.043
Food consumption rate-CKvsAI-UP	Equal variances assumed	1.247	0.327	-6.685	4	0.003
	Equal variances not assumed			-6.685	2.575	0.011
Food consumption rate-CKvsTI-UP	Equal variances assumed	5.19	0.085	-4.468	4	0.011
	Equal variances not assumed			-4.468	2.585	0.028

Food consumption rate-CKvsAI-FM	Equal variances assumed	0.07	0.804	-6.057	4	0.004
	Equal variances not assumed			-6.057	3.92	0.004
Food consumption rate-CKvsTI-FM	Equal variances assumed	4.688	0.096	-3.493	4	0.025
	Equal variances not assumed			-3.493	2.641	0.048
Food consumption rate-CKvsAI-MA	Equal variances assumed	3.897	0.12	-4.301	4	0.013
	Equal variances not assumed			-4.301	2.542	0.032
Food consumption rate-CKvsTI-MA	Equal variances assumed	2.86	0.166	-5.958	4	0.004
	Equal variances not assumed			-5.958	2.767	0.012
Food consumption rate-CKvsAI-JM	Equal variances assumed	0.241	0.649	-3.06	4	0.038
	Equal variances not assumed			-3.06	3.896	0.039
Food consumption rate-CKvsTI-JM	Equal variances assumed	0.232	0.655	-4.584	4	0.01
	Equal variances not assumed			-4.584	3.816	0.011
Food consumption rate-CKvsAI-BP	Equal variances assumed	2.821	0.168	-3.023	4	0.039
	Equal variances not assumed			-3.023	2.872	0.06
Food consumption rate-CKvsTI-BP	Equal variances assumed	0.698	0.451	-3.63	4	0.022
	Equal variances not assumed			-3.63	3.188	0.033
Food consumption rate-CKvsAI-AK	Equal variances assumed	1.217	0.332	-4.604	4	0.01
	Equal variances not assumed			-4.604	3.186	0.017
Food consumption rate-CKvsTI-AK	Equal variances assumed	0.799	0.422	-11.05	4	0
	Equal variances not assumed			-11.05	3.565	0.001
Food consumption rate-CKvsAI-TA	Equal variances assumed	3.582	0.131	-6.487	4	0.003
	Equal variances not assumed			-6.487	2.449	0.013

Food consumption rate-CKvsTI-TA	Equal variances assumed	5.166	0.085	-14.929	4	0
	Equal variances not assumed			-14.929	2.143	0.003
Total protein-CKvsAI-UP	Equal variances assumed	2.041	0.226	3.46	4	0.026
	Equal variances not assumed			3.46	2.29	0.061
Total protein-CKvsTI-UP	Equal variances assumed	0.047	0.839	2.838	4	0.047
	Equal variances not assumed			2.838	3.923	0.048
Total protein-CKvsAI-FM	Equal variances assumed	0.079	0.792	3.093	4	0.036
	Equal variances not assumed			3.093	3.931	0.037
Total protein-CKvsTI-FM	Equal variances assumed	0.168	0.703	3.541	4	0.024
	Equal variances not assumed			3.541	3.726	0.027
Total protein-CKvsAI-MA	Equal variances assumed	4.087	0.113	4.062	4	0.015
	Equal variances not assumed			4.062	2.703	0.033
Total protein-CKvsTI-MA	Equal variances assumed	4.108	0.113	2.945	4	0.042
	Equal variances not assumed			2.945	2.713	0.068
Total protein-CKvsAI-JM	Equal variances assumed	1.294	0.319	-5.963	4	0.004
	Equal variances not assumed			-5.963	2.905	0.01
Total protein-CKvsTI-JM	Equal variances assumed	1.204	0.334	-6.35	4	0.003
	Equal variances not assumed			-6.35	3.337	0.006
Total protein-CKvsAI-BP	Equal variances assumed	5.705	0.075	-2.747	4	0.052
	Equal variances not assumed			-2.747	2.31	0.095
Total protein-CKvsTI-BP	Equal variances assumed	3.039	0.156	-3.011	4	0.04
	Equal variances not assumed			-3.011	2.347	0.078

Total protein-CKvsAI-AK	Equal variances assumed	3.393	0.139	-4.252	4	0.013
	Equal variances not assumed			-4.252	2.652	0.03
Total protein-CKvsTI-AK	Equal variances assumed	2.019	0.228	-2.767	4	0.05
	Equal variances not assumed			-2.767	3.221	0.064
Total protein-CKvsAI-TA	Equal variances assumed	1.423	0.299	-1.072	4	0.344
	Equal variances not assumed			-1.072	3.29	0.356
Total protein-CKvsTI-TA	Equal variances assumed	4.724	0.095	-1.665	4	0.171
	Equal variances not assumed			-1.665	2.462	0.214
Total amino acids-CKvsAI-UP	Equal variances assumed	1.342	0.311	-6.016	4	0.004
	Equal variances not assumed			-6.016	2.563	0.014
Total amino acids-CKvsTI-UP	Equal variances assumed	8.833	0.041	-0.132	4	0.901
	Equal variances not assumed			-0.132	2.062	0.907
Total amino acids-CKvsAI-FM	Equal variances assumed	0.757	0.433	4.951	4	0.008
	Equal variances not assumed			4.951	3.347	0.012
Total amino acids-CKvsTI-FM	Equal variances assumed	0.244	0.647	3.497	4	0.025
	Equal variances not assumed			3.497	3.908	0.026
Total amino acids-CKvsAI-MA	Equal variances assumed	5.828	0.073	-3.874	4	0.018
	Equal variances not assumed			-3.874	2.082	0.057
Total amino acids-CKvsTI-MA	Equal variances assumed	0.224	0.661	-7.562	4	0.002
	Equal variances not assumed			-7.562	3.763	0.002
Total amino acids-CKvsAI-JM	Equal variances assumed	0.008	0.934	3.981	4	0.016
	Equal variances not assumed			3.981	3.974	0.017

Total amino acids-CKvsTI-JM	Equal variances assumed	11.868	0.026	5.983	4	0.004
	Equal variances not assumed			5.983	2.065	0.025
Total amino acids-CKvsAI-BP	Equal variances assumed	2.087	0.222	3.096	4	0.036
	Equal variances not assumed			3.096	3.135	0.05
Total amino acids-CKvsTI-BP	Equal variances assumed	4.977	0.09	2.769	4	0.05
	Equal variances not assumed			2.769	2.511	0.085
Total amino acids-CKvsAI-AK	Equal variances assumed	5.039	0.088	3.305	4	0.03
	Equal variances not assumed			3.305	2.469	0.06
Total amino acids-CKvsTI-AK	Equal variances assumed	0.239	0.651	3.379	4	0.028
	Equal variances not assumed			3.379	3.881	0.029
Total amino acids-CKvsAI-TA	Equal variances assumed	5.075	0.087	2.882	4	0.045
	Equal variances not assumed			2.882	2.472	0.08
Total amino acids-CKvsTI-TA	Equal variances assumed	1.406	0.301	5.149	4	0.007
	Equal variances not assumed			5.149	3.345	0.011
Glucose-CKvsAI-UP	Equal variances assumed	1.129	0.348	17.455	4	0
	Equal variances not assumed			17.455	3.139	0
Glucose-CKvsTI-UP	Equal variances assumed	0.066	0.81	10.442	4	0
	Equal variances not assumed			10.442	3.955	0.001
Glucose-CKvsAI-FM	Equal variances assumed	11.163	0.029	3.095	4	0.036
	Equal variances not assumed			3.095	2.046	0.088
Glucose-CKvsTI-FM	Equal variances assumed	0.861	0.406	3.18	4	0.034
	Equal variances not assumed			3.18	3.582	0.039

Glucose-CKvsAI-MA	Equal variances assumed	0.563	0.495	3.76	4	0.02
	Equal variances not assumed			3.76	3.539	0.025
Glucose-CKvsTI-MA	Equal variances assumed	1.179	0.339	4.852	4	0.008
	Equal variances not assumed			4.852	3.484	0.012
Glucose-CKvsAI-JM	Equal variances assumed	0.072	0.802	5.975	4	0.004
	Equal variances not assumed			5.975	3.866	0.004
Glucose-CKvsTI-JM	Equal variances assumed	0.279	0.625	4.909	4	0.008
	Equal variances not assumed			4.909	3.505	0.011
Glucose-CKvsAI-BP	Equal variances assumed	2.02	0.228	7.386	4	0.002
	Equal variances not assumed			7.386	2.825	0.006
Glucose-CKvsTI-BP	Equal variances assumed	4.707	0.096	2.917	4	0.043
	Equal variances not assumed			2.917	2.262	0.087
Glucose-CKvsAI-AK	Equal variances assumed	0.177	0.695	4.589	4	0.01
	Equal variances not assumed			4.589	3.78	0.012
Glucose-CKvsTI-AK	Equal variances assumed	1.944	0.236	2.975	4	0.041
	Equal variances not assumed			2.975	3.194	0.054
Glucose-CKvsAI-TA	Equal variances assumed	4.436	0.103	7.242	4	0.002
	Equal variances not assumed			7.242	2.601	0.009
Glucose-CKvsTI-TA	Equal variances assumed	2.878	0.165	3.197	4	0.033
	Equal variances not assumed			3.197	2.902	0.052
Trehalose-CKvsAI-UP	Equal variances assumed	1.31	0.316	19.254	4	0
	Equal variances not assumed			19.254	2.673	0.001

Trehalose-CKvsTI-UP	Equal variances assumed	0.036	0.86	16.022	4	0
	Equal variances not assumed			16.022	3.846	0
Trehalose-CKvsAI-FM	Equal variances assumed	1.857	0.245	7.701	4	0.002
	Equal variances not assumed			7.701	3.103	0.004
Trehalose-CKvsTI-FM	Equal variances assumed	0.046	0.841	10.564	4	0
	Equal variances not assumed			10.564	3.803	0.001
Trehalose-CKvsAI-MA	Equal variances assumed	2.672	0.177	11.152	4	0
	Equal variances not assumed			11.152	2.481	0.004
Trehalose-CKvsTI-MA	Equal variances assumed	0.804	0.421	6.918	4	0.002
	Equal variances not assumed			6.918	3.322	0.004
Trehalose-CKvsAI-JM	Equal variances assumed	2.348	0.2	4.715	4	0.009
	Equal variances not assumed			4.715	2.338	0.031
Trehalose-CKvsTI-JM	Equal variances assumed	5.875	0.072	4.027	4	0.016
	Equal variances not assumed			4.027	2.189	0.048
Trehalose-CKvsAI-BP	Equal variances assumed	1.011	0.372	4.335	4	0.012
	Equal variances not assumed			4.335	3.292	0.019
Trehalose-CKvsTI-BP	Equal variances assumed	4.757	0.095	2.702	4	0.054
	Equal variances not assumed			2.702	2.533	0.089
Trehalose-CKvsAI-AK	Equal variances assumed	0.258	0.638	3.885	4	0.018
	Equal variances not assumed			3.885	3.599	0.022
Trehalose-CKvsTI-AK	Equal variances assumed	3.978	0.117	7.726	4	0.002
	Equal variances not assumed			7.726	2.429	0.009

Trehalose-CKvsAI-TA	Equal variances assumed	1.257	0.325	7.205	4	0.002
	Equal variances not assumed			7.205	2.639	0.008
Trehalose-CKvsTI-TA	Equal variances assumed	0.576	0.49	6.382	4	0.003
	Equal variances not assumed			6.382	3.162	0.007
Free fatty acids -CKvsAI-UP	Equal variances assumed	2.066	0.224	-2.957	4	0.042
	Equal variances not assumed			-2.957	3.16	0.056
Free fatty acids -CKvsTI-UP	Equal variances assumed	5.711	0.075	-3.562	4	0.024
	Equal variances not assumed			-3.562	2.49	0.051
Free fatty acids -CKvsAI-FM	Equal variances assumed	4.307	0.107	-2.821	4	0.048
	Equal variances not assumed			-2.821	2.706	0.075
Free fatty acids -CKvsTI-FM	Equal variances assumed	2.786	0.17	-3.071	4	0.037
	Equal variances not assumed			-3.071	2.874	0.058
Free fatty acids -CKvsAI-MA	Equal variances assumed	0.493	0.521	-12.727	4	0
	Equal variances not assumed			-12.727	3.755	0
Free fatty acids -CKvsTI-MA	Equal variances assumed	0.393	0.565	-12.841	4	0
	Equal variances not assumed			-12.841	3.816	0
Free fatty acids -CKvsAI-JM	Equal variances assumed	1.711	0.261	-5.233	4	0.006
	Equal variances not assumed			-5.233	3.244	0.011
Free fatty acids -CKvsTI-JM	Equal variances assumed	2.231	0.21	-3.86	4	0.018
	Equal variances not assumed			-3.86	2.493	0.042
Free fatty acids -CKvsAI-BP	Equal variances assumed	1.08	0.357	13.393	4	0
	Equal variances not assumed			13.393	2.668	0.002

Free fatty acids -CKvsTI-BP	Equal variances assumed	3.347	0.141	3.62	4	0.022
	Equal variances not assumed			3.62	2.46	0.05
Free fatty acids -CKvsAI-AK	Equal variances assumed	2.334	0.201	5.218	4	0.006
	Equal variances not assumed			5.218	2.183	0.029
Free fatty acids -CKvsTI-AK	Equal variances assumed	0.34	0.591	3.349	4	0.029
	Equal variances not assumed			3.349	3.884	0.03
Free fatty acids -CKvsAI-TA	Equal variances assumed	0.975	0.379	5.022	4	0.007
	Equal variances not assumed			5.022	2.835	0.017
Free fatty acids -CKvsTI-TA	Equal variances assumed	0.541	0.503	3.235	4	0.032
	Equal variances not assumed			3.235	3.662	0.036
